# A Comparison of Two Nights of Ambulatory Sleep Testing in Arrhythmia Patients

**DOI:** 10.1155/2018/2394146

**Published:** 2018-06-03

**Authors:** Asmaa M. Abumuamar, Paul Dorian, David Newman, Colin M. Shapiro

**Affiliations:** ^1^Institute of Medical Science, Faculty of Medicine, University of Toronto, Toronto, ON, Canada; ^2^Department of Cardiology, St. Michael's Hospital, University of Toronto, Toronto, ON, Canada; ^3^Department of Cardiology, Sunnybrook Health Sciences Centre, University of Toronto, Toronto, ON, Canada; ^4^Department of Psychiatry, Toronto Western Hospital, University Health Network, University of Toronto, Toronto, ON, Canada

## Abstract

**Introduction:**

Obstructive sleep apnea (OSA) is common and usually underdetected in patients with cardiac arrhythmia. Ambulatory sleep testing may provide an alternative method for detection of OSA under realistic conditions compared to in-laboratory polysomnography. We aimed to (1) determine the sleep architecture in arrhythmia patients; (2) detect differences in sleep parameters between patients with and without OSA; and (3) compare the results of two consecutive nights of unattended ambulatory sleep testing.

**Methods:**

Consecutive patients with unknown OSA status were recruited from arrhythmia clinics. Patients underwent two consecutive nights of self-applied in-home sleep testing replete with electroencephalogram (EEG) recording.

**Results:**

One hundred patients were recruited. The mean age was 64 ± 13 years (70% males). OSA (AHI ≥ 5/h) was detected in 85% of patients. In the total sample, the sleep efficiency was reduced, and sleep onset latency was longer compared to a reference population of the same age. In patients with OSA, the sleep efficiency and the percentage of slow wave sleep were reduced; however, the arousal and periodic limb movement indices were increased compared to patients without OSA. The two nights of the ambulatory sleep testing showed consistent results with an excellent test-retest reliability for the AHI (ICC = 0.813). REM latency was shorter during the second night of sleep recording (*p* = 0.02). There were no other significant differences in the sleep architecture, respiratory indices, and other sleep parameters between the first and the second night of the ambulatory sleep recording.

**Conclusions:**

There is no significant difference in the respiratory parameters obtained during two consecutive nights of ambulatory sleep testing. Ambulatory studies incorporating EEG may provide a reliable, convenient, and economically efficient method for sleep assessment and there appears to be no significant night-to-night variability.

## 1. Introduction

Obstructive sleep apnea (OSA) is a common disorder associated with significant cardiovascular morbidities [1, 2]. OSA has been identified as a risk factor for the induction and progression of significant cardiac rhythm disturbance including atrial fibrillation [3]. The diagnosis and treatment of OSA may decrease the recurrence and/or severity of cardiac arrhythmia [3]. The gold-standard method for the diagnosis of OSA is an overnight in-laboratory polysomnography (PSG) [4]. The severity of OSA is classified according to the apnea-hypopnea-index (AHI), which is the number of obstructive events per hour of sleep. OSA is considered mild with an AHI between 5 and 15/hour; moderate with an AHI of 15 and 30/hour; and severe with an AHI ≥ 30/hour [5]. Other measures of the disease activity include the degree of hypoxemia and the arousal index. For some, the presence of the clinical sequelae of apnea is considered when evaluating the clinical severity of OSA. These may include excessive daytime sleepiness, hypertension, and depression.

The overnight in-laboratory PSG involves the concurrent recording of physiologic signals during sleep, including the electroencephalogram (EEG), electrooculogram (EOG), electromyogram (EMG), and electrocardiogram (ECG). For identification of obstructive events, an oronasal airflow sensor is used to monitor the airflow. For monitoring of respiratory effort, dual thoracoabdominal belts are used. Typically, oxygen saturation is measured using a pulse oximeter attached to the patient's finger. In addition, a nasal pressure transducer is used for snoring detection.

The in-laboratory PSG has several challenges including significant financial, human, and healthcare costs [6]. Polysomnographic studies are typically conducted in a sleep laboratory which is an unfamiliar environment for patients and is associated with the discomfort of being connected to a recording device with several wires. These environmental changes create a phenomenon known as the “first night effect” [7], which implicates changes in sleep quality and efficiency resulting in inaccurate and unrepresentative results. Moreover, there are differences in the recording techniques among different sleep laboratories, and until recently a lack of awareness of the implication of rapid eye movement (REM) and nonrapid eye movement (NREM) related apneas [8]. In addition, the inconsistency of the results related to different criteria, such as the threshold of oxygen desaturation, exists between different laboratories. Some variability in sleep data may result due to night-to-night variability or the “first night effect” [7]. Further, in-laboratory PSG requires monitoring by an attending sleep technician. Another challenge is the scarcity and distribution of sleep laboratories compared to the high number of patients who may need a sleep assessment such as arrhythmia patients. Finally, a sleep study may need to be repeated if inadequate sleep was recorded as in such case inadequate data may not be reliable for obtaining a diagnosis. The previous reasons suggest that in-laboratory PSG increases the healthcare burden and could be a contributing factor to the underrecognition of OSA.

Portable sleep monitors provide an alternative method for sleep assessment [9]. Several models of portable monitors have emerged in the sleep market including devices that record only snoring, devices that record airflow and breathing, while other devices combine breathing and EEG recording. However, a reliable sleep assessment would require EEG monitoring. The EEG recording provides the sleep architecture, a sine qua non to establishing the duration of REM sleep, the total sleep time, and intervening awakenings. These parameters are necessary for an accurate estimation of the AHI, which indicates the severity of OSA. For example, patients with OSA and insomnia may have incorrect results due to inaccurate total sleep time assessment. EEG recording also allows the detection of REM sleep and a separate evaluation of REM-related apneas which may be critical for an effective treatment. Further, hypopneas without accompanied oxygen desaturation, however, followed by arousal from sleep are not considered by devices that only detect apneas, which may underestimate the severity of OSA. According to the standard American Academy of Sleep Medicine (AASM) criteria, these hypopneas should be part of the AHI.

In this study, we aimed to perform a full sleep assessment for two consecutive nights by ambulatory sleep monitor that includes EEG recording. In addition, we aimed to determine any significant differences in the sleep architecture and other sleep parameters between patients with and without OSA and to compare the results of the two consecutive nights of ambulatory sleep testing to detect any significant night-to-night variability. Finally, we aimed to determine the reliability of a self-applied portable sleep test and its application in arrhythmia clinics, which may facilitate an early detection of OSA.

## 2. Methods

### 2.1. Study Design

This is a prospective cohort study involving patients with cardiac arrhythmia. Ethical approval for the study was obtained from the University Health Network and from St. Michaels Hospital Research Ethics Boards. Nonselected consecutive patients were recruited from two major specialized arrhythmia clinics in Toronto, ON, Canada. Adult patients without a previous diagnosis and/or treatment of OSA were included. Patients who had had a sleep study within six months prior to recruitment were excluded. There were no other exclusion criteria. Participants underwent two consecutive nights of self-applied ambulatory sleep testing using a home sleep monitor that provides a full sleep assessment including EEG recording.

### 2.2. Ambulatory Sleep Testing

Participants underwent two consecutive nights of self-applied unattended ambulatory sleep recording using the Somté PSG (v2) sleep monitor (P/N: 8023-0001-02, Compumedics Limited, Australia). Physiological signals were acquired by electrodes and sensors which are connected between the patient and the sleep monitor. Patients received full instructions regarding the correct application of electrodes and sensors. In addition, patients were provided with a simplified manual enriched with colored pictures to demonstrate all steps they need to follow in their home.

The sleep recording involved a full sleep assessment including EEG, EOG, EMG, ECG, airflow, snore, respiratory effort, oxygen saturation, body position, and pulse rate and pulse waveform. A simple system for EEG monitoring was designed by the Neurozone MSH Inc., Canada. The EEG system used in this study allows for identical scoring as the standard polysomnography (PSG). M1 and M2 electrodes were placed over the left and right mastoid process, respectively. FZ, a reference for M1 and M2, was placed in the middle of the forehead. Left and right standard EOG channels were applied. The EOG channels were referenced to the contralateral mastoid site (M1 and M2). In addition, two standard chin EMG electrodes were applied over the mentalis and submentalis muscles. For leg muscle activity, two standard limb movement sensors were placed on each leg. The limb sensors were placed symmetrically, two centimeters apart, over the tibialis anterior muscle. Modified Limb II and V1 standard ECG electrodes were applied. A nasal pressure transducer was used to monitor airflow and snore. Dual thoracoabdominal respiratory inductance plethysmography (RIP) sensors were used to monitor respiratory effort during the study. For monitoring oxygen saturation, we used a pulse oximeter probe with a maximum acceptable signal averaging time of ≤3 seconds at a heart rate of 80 beats per minutes. Moreover, the unit includes an internal position sensor that records patients' body position during sleep.

The ESPRIT NOVA client access (v1.1.402) software was utilized for automatic analysis of polysomnogram data. Further, the studies were manually scored, and the automated analysis was edited by a qualified sleep technologist. The scoring of each night was done independently, and scorers were blinded to patients' identification information and to the results of the first night of the home sleep test. The sleep studies were reviewed and approved by a sleep specialist. The standard AASM criteria for scoring and interpretation of PSG data were followed.

### 2.3. Statistical Analysis

Nonparametric Mann–Whitney test was used to compare means of the sleep parameters between patients with and without OSA. Nonparametric Wilcoxon Signed Ranks Test for matched pairs was used to detect differences between the first and second nights of ambulatory sleep testing. Bland-Altman plot was constructed to investigate the existence of any systematic bias between the overall AHI of the first and second night and to identify possible outliers. One sample *t*-test was used to detect if there is a significant difference between the two measurements (AHI1 and AHI2). A linear regression analysis was performed to determine if there is a linear trend of proportional bias above or below the mean difference line of Bland-Altman plot. The difference between AHI1 and AHI2 was the dependent variable and the mean AHI was the independent variable. The test-retest reliability was assessed using the interclass correlation coefficient (ICC) based on two-way random effects model for single measures (absolute agreement).

## 3. Results

### 3.1. Population Characteristics

One hundred patients with atrial fibrillation were recruited. The mean age of patients was 64 ± 13 years. Seventy percent were males and 30% were females. The mean body-mass-index (BMI) was 29 ± 6 kg/m^2^ (range: 17–47). Obesity defined as a BMI ≥ 30 kg/m^2^ was present in 33% of the study sample. The mean neck circumference was 39.7 ± 3.7 cm. Forty-nine percent of the group were hypertensive and 11% were diabetics. Twenty percent had a previous ablation for atrial fibrillation/flutter. Eighty-five percent of patients (*n* = 95) had a score ≥ 3 on the STOP-BANG questionnaire. Twenty-two percent of patients (*n* = 85) had scores suggestive of excessive daytime sleepiness on the Epworth sleepiness scale. Of the total 100 patients, seventy-nine patients had two consecutive successful home sleep studies, while 21 patients had only one successful sleep study. An average AHI of the two nights was calculated for patients who had two recordings. For patients with one successful sleep study, the AHI obtained from that night was used. Of the total patients (*n* = 100), 85% (21 females and 64 males) had an AHI ≥ 5/hour of sleep. Fifty-five percent of patients had moderate and severe OSA (AHI ≥ 15/hour of sleep).

### 3.2. Sleep Parameters for the Study Population

#### 3.2.1. The Results of the Ambulatory Sleep Testing

*Sleep Macro-Architecture*. The nonparametric Mann–Whitney test revealed a significant difference in the sleep efficiency and the percentage of stage 3 NREM between patients with and without OSA. It is notable that the percentage of slow wave sleep in both OSA and non-OSA patients is substantially higher than one might expect in a population of this age. One might speculate that this may in part account for the nonclinical presentation in these patients with OSA. No other differences in the sleep macro-architecture were found between the two groups (Table 1). The respiratory indices, heart rate, arousal, and limb movement indices are shown in Table 2. During the second night of the ambulatory sleep testing, the sleep efficiency was reduced for the whole group. REM latency was normal. Sleep latency was slightly increased for the whole group. The nonparametric Mann–Whitney test revealed no significant differences in sleep macro-architecture between patients with and without OSA. However, there was a significant difference in the arousal index, PLMS, and PLMS index between the two groups. The results of the second night of the ambulatory sleep testing for patients with and without OSA are shown in supplementary Tables  1 and 2.

### 3.3. A Comparison between Two Nights of The Ambulatory Sleep Testing

The nonparametric Wilcoxon Signed Ranks Test for matched pairs showed no significant differences in the sleep parameters obtained from the two consecutive nights of the ambulatory sleep testing. The only difference was in REM latency (*P* = 0.02). There were no significant differences in the macro-architecture, respiratory events, heart rate, arousal, or limb movements (Table 3).

**Figure 1:**
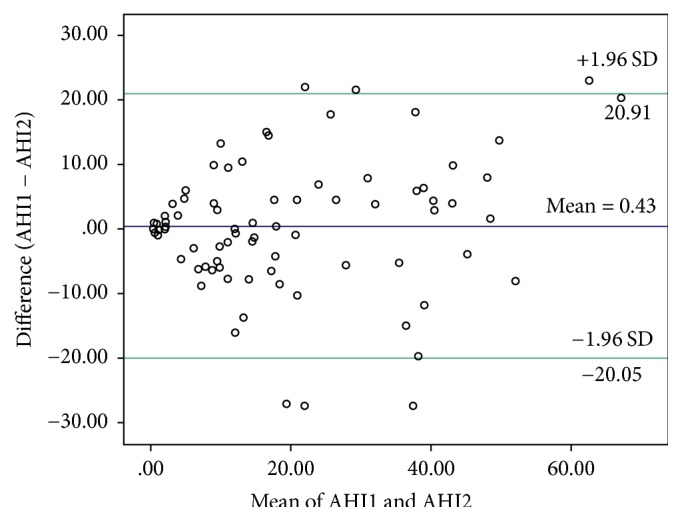
Bland-Altman plot shows the agreement between the apnea-hypopnea-index obtained from the first and second night of the ambulatory sleep testing. AHI: apnea-hypopnea-index; SD: standard deviation.

Bland-Altman plot shows no systematic bias between the overall AHI of the first and second night of the ambulatory sleep testing (Figure 1). One sample *t*-test was showed no significant difference between the AHI of the two nights. The mean of the difference between the two AHI measurements was 0.43, SD = 10.45, and *P* = 0.7. A linear regression analysis showed that there is no proportional bias above or below the mean difference line of Bland-Altman plot (*t* = 1.65, *P* = 0.1). The results of the interclass correlation coefficient (ICC) two-way random effects model for single measures (absolute agreement) are shown in Table 3. The ICC values range from fair to excellent reliability. In particular, the ICC for the AHI was 0.813 indicating excellent test-retest reliability.

## 4. Discussion

In this study, we applied a novel technique of self-applied unattended ambulatory sleep testing in a group of patients with atrial fibrillation. The home sleep studies involved a full sleep assessment including adequate EEG recording. Analysis of the sleep data showed a reduced sleep efficiency in all participants compared to the standard reference for similar age group, indicating a high level of sleep disruption among the study population. REM latency was normal, while sleep onset latency was slightly increased for the whole group. Patients with OSA had a significantly lower sleep efficiency and a shorter percentage of stage 3 compared to patients without OSA. Patients with OSA also had a higher arousal index, PLMS, and PLMS index compared to people without OSA. The results of the two nights showed a good agreement with no significant differences in the macro-architecture, respiratory events, heart rate, arousal, or limb movements between the first and the second night of the ambulatory sleep testing. The only significant difference was in REM latency, which was decreased during the second night. The results showed an excellent test-retest reliability for diagnosis of OSA.

In-laboratory PSG studies are associated with a phenomenon known as “the first night effect”. The characteristic differences in the sleep architecture on the first night as compared to subsequent nights may contribute to misdiagnoses of 15–25% of first night sleep studies done in a sleep laboratory [10]. First night effects include decreased total sleep time, sleep efficiency, and REM sleep [11]. In addition, the first night effect causes a delay in the onset of slow wave sleep and REM sleep [11]. The perception has been that the first night effects occur as a combination of the change in environment and the extensive wiring to the sleep equipment. In our study, the application of a home sleep testing provided consistent results with insignificant night-to-night variability. This may imply that the location is key. The advantage of the unattended ambulatory sleep testing includes sleep assessment in a convenient home environment. In addition, there was no extensive wiring compared to the overnight polysomnography recorded in a laboratory. The ambulatory sleep testing involved a small device with the minimum number of electrodes that provide a full sleep assessment, which may detect most sleep disorders. The ambulatory recording provided an estimation of the sleep architecture, the total sleep time, and intervening awakenings, which are important for an accurate estimation of the AHI. The diagnosis of OSA along with full data of sleep architecture is essential as this provides the advantage of detection of apnea during REM sleep.

Further, the automatic analysis of the study data provides immediate results within 48 hours, which may reduce the waiting time for diagnostic sleep studies. There are a large number of patients with cardiac arrhythmia and other cardiovascular disorders who need sleep assessment for OSA. The application of the ambulatory sleep testing in these populations may help in early detection and treatment of OSA. Moreover, home sleep testing may eliminate the geographical barriers, high costs, and the variability of sleep results associated with in-laboratory PSG studies. The ambulatory sleep testing may permit an equitable access of individuals to sleep care. Therefore, implementation of these devices could solve some of the challenges associated with obtaining in-laboratory PSG studies. The limitations of our study may include selection and observational bias. The AASM recommends the use of a nasal pressure transducer (with or without square root transformation of the signal) for identification of hypopneas and as an alternative to oronasal thermal sensors for detection of apneas. We used a nasal pressure transducer (with square root transformation of the signal) to monitor airflow, which is limited to nasal airflow leaving mouth flow undetected. The use of both oronasal thermal sensors and nasal pressure devices may be more accurate than either one.

## 5. Conclusion

In conclusion, a technically adequate unattended ambulatory sleep monitoring with full sleep assessment may provide an affordable and practical solution that may aid in early detection and treatment of OSA and several sleep disorders. This opens the prospect of sleep evaluation as a front-line medical service rather than a tertiary clinical service and allows family physicians, for example, to become involved in performing this important health metric.

## Figures and Tables

**Table 1:** Sleep macro-architecture for the study sample during first night of ambulatory recording.

	Total (*N* = 92)	OSA (*N* = 77)	Non-OSA (*n* = 15)	Difference
Sleep Onset Latency (min)	45.3 ± 50	48 ± 54	32.6 ± 20	*P* = 0.4
Sleep Efficiency (%)	72 ± 18.3	70 ± 19	81 ± 12.4	*P* = 0.02^*∗*^
Stage 1 (%)	5.3 ± 6	5.4 ± 6	4.8 ± 4.4	*P* = 0.9
Stage 2 (%)	59.6 ± 14.4	61 ± 14	52.7 ± 14.5	*P* = 0.06
Stage 3 (%)	11 ± 10.7	9.8 ± 9.9	17.1 ± 13	*P* = 0.02^*∗*^
Stage 4 (%)	4.5 ± 8.2	3.8 ± 7.6	8 ± 10.4	*P* = 0.1
REM (%)	18 ± 9.4	18 ± 9.8	18.7 ± 7.5	*P* = 0.5
REM Latency (min)	102.3 ± 65.6 (*n* = 89)	97 ± 57.8 (*n* = 74)	128 ± 93.5 (*n* = 15)	*P* = 0.4

Values are expressed as mean ± SD. Nonparametric test was used to compare means of groups. *∗* indicates significance. *N* = number; OSA: obstructive sleep apnea; REM: rapid eye movement.

**Table 2:** Respiratory and other sleep parameters for the study sample during first night of ambulatory sleep testing.

	Total (*n* = 92)	OSA (*n* = 77)	Non-OSA (*n* = 15)	Difference
AHI/h	19.55 ± 17	22.90 ± 16.64	2.34 ± 2	*P* = 0.000^*∗*^
Supine AHI/h	24 ± 26.96	27.74 ± 27.81	5.20 ± 8.52	*P* = 0.001^*∗*^
Non-supine AHI/h	15.56 ± 17.80	18.38 ± 18.15	1.07 ± 1.43	*P* = 0.000^*∗*^
REM AHI/h	25.67 ± 24.42	30.08 ± 24.37	3.32 ± 3.5	*P* = 0.000^*∗*^
NREM-AHI/h	18.18 ± 17	21.30 ± 16.88	2.17 ± 1.90	*P* = 0.000^*∗*^
Minimum Saturation (%)	81 ± 13.3	79.67 ± 14	88.15 ± 3.3	*P* = 0.001^*∗*^
Average nocturnal heart rate bpm	61.81 ± 12.81	62.26 ± 13.34	59.53 ± 9.71	*P* = 0.3
Arousal Index/h	15.41 ± 8.62	15.66 ± 9	12.46 ± 5.86	*P* = 0.2
PLMS (count)	50.23 ± 96.65	51.46 ± 93.24	43.93 ± 116	*P* = 0.1
PLMS Index/h	10.20 ± 18.64	10.98 ± 19.27	6.20 ± 14.91	*P* = 0.2

Values are expressed as mean ± SD. Nonparametric Mann-Whitney test was used to compare means of groups. *∗* indicates significance. AHI: apnea-hypopnea-index; *N* = number; OSA: obstructive sleep apnea; REM: rapid eye movement; h: hour; bpm: beat per minute; PLMS: periodic limb movements in sleep.

**Table 3:** A comparison between sleep parameters of first and second night of ambulatory sleep testing.

	First night	Second night	Difference between means	ICC (2, 1)
Sleep Onset Latency (min)	45.29 ± 49.93	30.8 ± 27.85	*P* = 0.5	0.320
Sleep Efficiency (%)	71.91 ± 18.27	72.10 ± 15.84	*P* = 0.9	0.396
Stage 1 (%)	5.28 ± 5.61	5.82 ± 7.68	0.7	0.553
Stage 2 (%)	59.58 ± 14.36	57.53 ± 17.31	0.4	0.599
Stage 3 (%)	11.05 ± 10.68	12.79 ± 12.61	0.2	0.757
Stage 4 (%)	4.47 ± 8.16	3.34 ± 7.05	0.7	0.740
REM (%)	18.13 ± 9.38	17.12 ± 8.91	0.3	0.549
REM Latency (min)	102.32 ± 65.57	92.46 ± 80.90	0.02^*∗*^	0.379
AHI/h	19.55 ± 17	20.26 ± 17.23	*P* = 0.6	0.813
Supine AHI/h	24 ± 26.96	23.29 ± 24.65	*P* = 0.3	0.477
Non-supine AHI/h	15.56 ± 17.80	14.86 ± 18.34	*P* = 0.4	0.627
REM AHI/h	25.67 ± 24.42	23.34 ± 23.54	*P* = 0.1	0.540
NREM-AHI/h	18.18 ± 17	19 ± 17.5	*P* = 0.5	0.781
Minimum Saturation (%)	81 ± 13.3	83.24 ± 8.45	*P* = 0.4	0.325
Average nocturnal heart rate bpm	61.81 ± 12.81	62.26 ± 14.3	*P* = 0.7	0.695
Arousal Index/h	15.41 ± 8.62	14.81 ± 7.90	*P* = 0.9	0.618
PLMS (count)	50.23 ± 96.65	49.82 ± 99.83	*P* = 0.2	0.644
PLMS Index/h	10.20 ± 18.64	9.71 ± 18.47	*P* = 0.3	0.709

Values are expressed as mean ± SD. Nonparametric Wilcoxon signed rank test was used to compare means of groups. *∗* indicates significance. AHI: apnea-hypopnea-index; ICC: interclass correlation coefficient; OSA: obstructive sleep apnea; PLMS: periodic limb movements in sleep; REM: rapid eye movement; h: hour; bpm: beat per minute.

## Data Availability

There is a further component of the study ongoing, a follow-up concerning the impact of treatment. Once that is completed and published, data may be available upon request after obtaining permission from institutions and funder.

## Abbreviations

AASM: American Academy of Sleep Medicine
AHI: Apnea-hypopnea-index
BMI: Body-mass-index
ECG: Electrocardiogram
EEG: Electroencephalogram
EMG: Electromyogram
EOG: Electrooculogram
OSA: Obstructive sleep apnea
PSG: Polysomnography
REM: Rapid eye movement.

## Supplementary Materials

Supplementary Materials Supplementary Table 1: sleep macro-architecture for the study sample during second night of ambulatory recording. Supplementary Table  2: respiratory parameters for the study sample during second night of ambulatory sleep testing.
