# Supplementary material for: A Comparison of Two Nights of Ambulatory Sleep Testing in Arrhythmia Patients
Source: Sleep Disord. 2018 Jun 3;2018:2394146. doi: 10.1155/2018/2394146 (PMC6008611; doi:10.1155/2018/2394146)
Supplement: Supplementary Materials — Supplementary Table 1: sleep macro-architecture for the study sample during second night of ambulatory recording. Supplementary Table 2: respiratory parameters for the study sample during second night of ambulatory sleep testing. [file 2394146.f1.pdf]

Supplementary table 1: Sleep macro-architecture for the study sample during second night of ambulatory recording.

	**Total (N=87)**	**OSA (N=73)**	**Non-OSA (n=14)**	**Difference (Sig)**
**Sleep Onset Latency (min)**	30.8 ± 27.85	37.52 ± 28.12	41 ± 27.23	P=0.4
**Sleep Efficiency (%)**	72.10 ± 15.84	70.88 ± 16.26	78.49 ± 11.98	P= 0.1
**Stage 1 (%)**	5.82 ± 7.68	6.15 ± 8.16	4.12 ± 4.18	P=0.3
**Stage 2 (%)**	57.53 ± 17.31	58.39 ± 17.80	53 ± 14.1	P=0.2
**Stage 3 (%)**	12.79 ± 12.61	12.46 ± 13	14.48 ± 10.54	P=0.2
**Stage 4 (%)**	3.34 ± 7.05	1.95 ± 4.18	9.84 ± 12.64	P=0.07
**REM (%)**	17.12 ± 8.91	16.78 ± 9.17	18.87 ± 7.43	P=0.7
**REM Latency (min)**	92.46 ±80.90 (n=86)	89.62 ± 80.76 (n=72)	107 ± 83 (n=14)	P=0.5

Values are expressed as mean ± SD. Non-parametric test was used to compare means of groups. *indicates significance. N=number; OSA: obstructive sleep apnea; REM: rapid eye movement.

Supplementary table 2: Respiratory parameters for the study sample during second night of ambulatory sleep testing.

	**Total (n=87)**	**OSA (n=73)**	**Non-OSA (n=14)**	**Difference**
**AHI/h**	20.26 ± 17.23	23.88 ± 16.54	1.66 ± 1.63	P=0.000*
**Supine AHI/h**	23.29 ± 24.65	27.43 ± 24.90	1.99 ± 2	P=0.000*
**Non-supine AHI/h**	14.86 ± 18.34	17.59 ± 18.87	0.83 ± 1.1	P=0.000*
**REM AHI/h**	23.34 ± 23.54	27.22 ± 23.83	3.37 ± 2.68	P=0.000*
**NREM-AHI/h**	19 ± 17.5	22.44 ± 17.12	1.41 ± 2.06	P=0.000*
**Minimum Saturation (%)**	83.24 ± 8.45	82.23 ± 8.50	88.15 ± 3.3	P=0.001*
**Average nocturnal heart rate bpm**	62.26 ± 14.3	63 ± 14.85	58.28 ± 10.58	P=0.1
**Arousal Index/h**	14.81 ± 7.90	15.89 ± 7.97	9.2 ± 4.55	P=0.002*
**PLMS (count)**	49.82 ± 99.83	58.16 ± 106.87	6.35 ± 15.73	P=0.02*
**PLMS Index/h**	9.71 ± 18.47	11.33 ± 19.73	1.28 ± 3.31	P=0.01*

Values are expressed as mean ± SD. Non-parametric Mann Whitney test was used to compare means of groups. *indicates significance. AHI: apnea-hypopnea-index; N=number; OSA: obstructive sleep apnea; REM: rapid eye movement; h: hour; bpm: beat per minute; PLMS: periodic limb movements in sleep.
